# The stress polarity signaling (SPS) pathway serves as a marker and a target in the leaky gut barrier: implications in aging and cancer

**DOI:** 10.26508/lsa.201900481

**Published:** 2020-02-10

**Authors:** Pradipta Ghosh, Lee Swanson, Ibrahim M Sayed, Yash Mittal, Blaze B Lim, Stella-Rita Ibeawuchi, Marc Foretz, Benoit Viollet, Debashis Sahoo, Soumita Das

**Affiliations:** 1 Department of Medicine, University of California San Diego, La Jolla, CA, USA; 2 Department of Cellular and Molecular Medicine, University of California San Diego, La Jolla, CA, USA; 3 Moores Cancer Center at UC San Diego Health, La Jolla, CA, USA; 4 Veterans Affairs Medical Center, La Jolla, CA, USA; 5 Department of Pathology, University of California San Diego, La Jolla, CA, USA; 6 Institut National de la Santé et de la Recherche Médicale (French Institute of Health and Medical Research) (INSERM) U1016, Institut Cochin, Paris, France; 7 Centre National de la Recherche Scientifique (National Center for Scientific Research) (CNRS) United for Medical Research (UMR) 8104, Paris, France; 8 Université Paris Descartes, Sorbonne Paris Cité, Paris, France; 9 Department of Pediatrics, University of California San Diego, La Jolla, CA, USA; 10 Department of Computer Science and Engineering, Jacob’s School of Engineering, University of California San Diego, La Jolla, CA, USA; 11 Microbiology and Immunology Department, Assiut University, Asyut, Egypt

## Abstract

The gut barrier separates trillions of microbes from the largest immune system in the body; when compromised, a “leaky” gut barrier fuels systemic inflammation, which hastens the progression of chronic diseases. Strategies to detect and repair the leaky gut barrier remain urgent and unmet needs. Recently, a stress-polarity signaling (SPS) pathway has been described in which the metabolic sensor, AMP-kinase acts via its effector, GIV (also known as Girdin) to augment epithelial polarity exclusively under energetic stress and suppresses tumor formation. Using murine and human colon-derived organoids, and enteroid-derived monolayers (EDMs) that are exposed to stressors, we reveal that the SPS-pathway is active in the intestinal epithelium and requires a catalytically active AMP-kinase. Its pharmacologic augmentation resists stress-induced collapse of the epithelium when challenged with microbes or microbial products. In addition, the SPS-pathway is suppressed in the aging gut, and its reactivation in enteroid-derived monolayers reverses aging-associated inflammation and loss of barrier function. It is also silenced during progression of colorectal cancers. These findings reveal the importance of the SPS-pathway in the gut and highlights its therapeutic potential for treating gut barrier dysfunction in aging, cancer, and dysbiosis.

## Abstract (precis)

Using patient-derived organoids the authors show how a specialized polarity pathway protects our gut barrier from stress-induced collapse. Findings highlight both diagnostic and therapeutic potential of the pathway for treating gut barrier dysfunction in aging, cancer, and dysbiosis.

## Introduction

The gut is a complex environment; the gut mucosa maintains immune homeostasis in physiology by serving as a barrier that restricts access of trillions of microbes, diverse microbial products, food antigens, and toxins to the largest immune system in the body. The intestinal barrier is the largest mucosal surface that separates diverse stressors (trillions of microbes, toxins, and food antigens) on one side from the largest immune system on the other. A compromised gut barrier allows microbes and unwanted antigens to cross the epithelium and generate inflammation (systemic endotoxemia), which may contribute to a variety of diseases, ranging from metabolic syndrome and chronic organ dysfunctions to neurodegenerative diseases and cancers (Yacyshyn et al, 1996; Barbara, 2006; Camilleri & Gorman, 2007; Sandek et al, 2007, 2008, 2012; Alam et al, 2014; Bischoff et al, 2014; Nouri et al, 2014; Samsam et al, 2014; van De Sande et al, 2014; Clairembault et al, 2015; Lee et al, 2015; Buscarinu et al, 2016; Xue et al, 2016; Ghosh, 2017). Evidence also shows that aging-related genes, that is, the sirtuins (SIRTs1, 3, 6), are critical for the integrity of the gut barrier and for controlling inflammation in the gut (Akimova et al, 2014; Akbulut et al, 2015; Liu et al, 2017b; Zhang et al, 2018). Despite the traction and the discovery of plausible targets to strengthen the barrier, for example, myosin light-chain kinase (Cunningham & Turner, 2012), our knowledge of the underlying mechanism(s) that reinforce the barrier when faced with stressors is incomplete, and practical strategies for pharmacologic modulation of the gut barrier remains unrealized.

The primary factor preventing the free access of stressors to our immune cells is a single layer of polarized intestinal epithelial cells strung together in solidarity by cell–cell junctions. Loss of cell polarity and junctions not only impairs organ development and function but can also serve as one of the first triggers for oncogenesis (Martin-Belmonte & Perez-Moreno, 2012). Establishment, maintenance (at baseline), and augmentation (upon stress) of epithelial barriers are achieved by signaling pathways that regulate polarization of epithelial cells. Epithelial polarity is established and maintained by a set of evolutionarily conserved signaling pathways whose integration in space and time dictates overall epithelial morphogenesis (St Johnston & Sanson, 2011), for example, CDC42 and PAR proteins, such as the PAR3-PAR6-aPKC polarity complex (Wodarz & Nathke, 2007) and pathways that regulate membrane exocytosis and lipid modifications (Wodarz & Nathke, 2007; St Johnston & Ahringer, 2010). Augmentation of epithelial polarity requires an additional signaling component which is triggered exclusively in response to stress. Three studies (Zhang et al, 2006; Lee et al, 2007; Zheng & Cantley, 2007) reported a role of AMP-activated protein kinase (AMPK) in the maintenance of epithelial cell polarity and barrier functions in the context of stress; who or what was its effector at the cell–cell junctions remained unknown. A decade later, Aznar et al (2016) demonstrated that GIV (G-α interacting vesicle–associated protein, also known as Girdin), a multimodular polarity scaffold protein is a substrate of AMPK and defined the molecular mechanisms by which the AMPK-GIV signaling axis protects the epithelium by stabilizing tight junctions (TJs) and preserving cell polarity when challenged with energetic stress. Using MDCK cells as a model of polarized mammalian cells, Aznar et al (2016) showed that energetic stress triggers localized activation of AMPK at the tricellular TJs which mark the most vulnerable cell–cell contacts in sheets of polarized cells. A significant part of the junction-stabilizing effects of AMPK agonists such as 5-Aminoimidazole-4-carboxamide ribonucleotide (AICAR) and metformin during energetic stress (Zhang et al, 2006; Zheng & Cantley, 2007) appeared to be mediated by AMPK via its downstream effector, pS245-GIV (Aznar et al, 2016). Based on these findings, the AMPK → pS245 GIV signaling axis was named the “Stress Polarity Signaling (SPS)-Pathway.” It was shown that the SPS-pathway inhibits oncogenic transformation and anchorage-independent growth of cancer cells; disruption of this pathway via mutations that make GIV non-phosphorylatable helps tumor cells escape such inhibition and gain proliferative advantage. These findings led to the conclusion that the SPS-pathway integrates stress-sensing and augmention of cell polarity pathways to serve as a deterrent to oncogenesis.

Despite these insights, the pathophysiologic relevance of such integration illustrated exclusively in cell lines, remained unclear. Here, we used a near-physiologic model system (co-cultures of microbes with mouse and human organoids) to assess the importance of this pathway in the gut lining. Our studies reveal the physiologic importance of the SPS-pathway in the maintenance of the gut barrier function and consequences when it is dysregulated. Findings also reveal the therapeutic potential of AMPK agonists such as metformin in strengthening the gut barrier. Over the years, the beneficial (protective) effect of multiple nutritional components, dietary supplements, and pharmacologic agents, including the widely prescribed AMPK-activator, metformin, on intestinal permeability in health and disease has been investigated; all studies converge on AMPK activation as a common pre-requisite for rendering such protection (reviewed in (Ghosh (2017)). Taken together with these prior works, our findings provide an impetus for harnessing the diagnostic and therapeutic potential of the SPS-pathway in the setting of a variety of diseases that are associated with increased intestinal permeability such as inflammaging and cancer.

## Results and Discussion

### The SPS-pathway is active in the colon epithelium and requires the catalytic activity of AMPK

**Figure 1.**
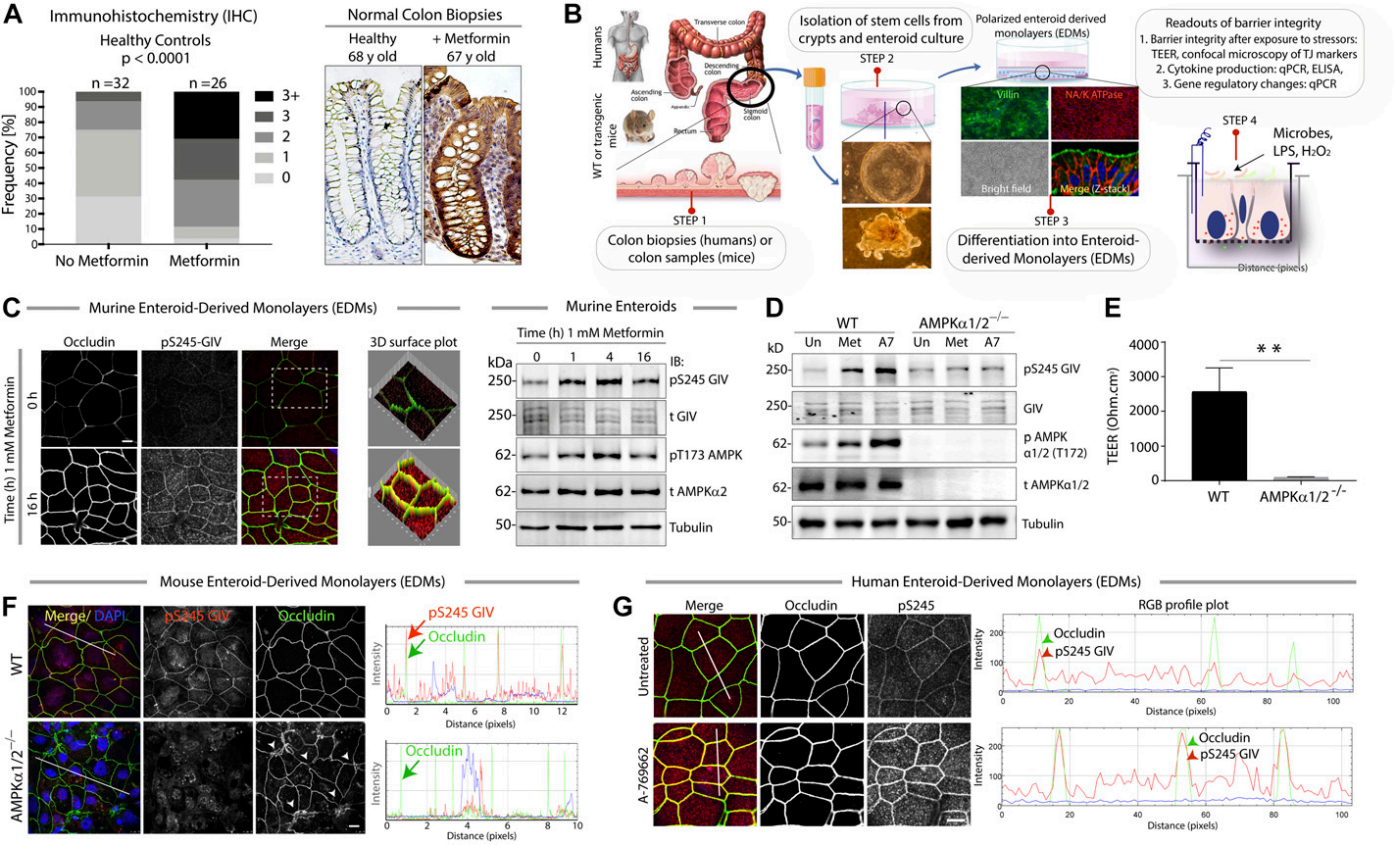
The stress polarity signaling (SPS) pathway is active in the gut lining, and its pharmacologic augmentation requires the catalytic activity of AMP-kinase. **(A)** *Left*: The SPS-pathway was evaluated in normal adult colon by immunohistochemistry on FFPE colonic biopsies using anti-pS245-GIV, and the staining intensity in the epithelium was scored (see Fig S1). Bar graph displays the proportion of patients in each group with varying intensities of staining. Two-sided Fisher’s exact test was used to calculate significance. *Right*: Representative images are presented from healthy adult without metformin (lowest “0” staining) and with metformin (highest “>3+” staining) within the cohort. **(B)** Schematic showing the key steps involved during the development of the stem cell-based organoid model, “gut-in-a-dish.” Fresh biopsies obtained from the colons of mice and humans (STEP 1) are used as source of stem cells to grow organoids (STEP 2). Organoids are differentiated into polarized enteroid-derived monolayers (EDMs; STEP 3) for co-culture studies with microbes and microbial products (exposed apical surface) to mimic the gut lumen in physiology and enable the assessment of barrier integrity (STEP 4). **(C)** *Left*: Murine EDMs treated with metformin for 0, 1, 4, and 16 h were fixed; stained with anti-pS245-GIV (red; a surrogate measure of the SPS-pathway), occludin (green; a *bonafide* tight junction [TJ] marker), and nucleus (DAPI); and analyzed by confocal microscopy. Images (left) display the findings at 0 and 16 h; see Fig S2 for all intermediate time points and for quantification of pixel intensity of occludin staining at TJs. Boxed areas were analyzed by 3D surface plot (Image J). Scale bars = 10 μm. *Right*: Immunoblots (right) on whole cell lysates of murine enteroids treated for 0, 1, 4, and 16 h incubated with anti-phospho(p) and anti-total(t) GIV and anti-AMPK proteins, and α-tubulin (as loading control) confirm activation of the SPS pathway in murine enteroids. Images and immunoblots presented are representative of ∼3–5 independent experiments. **(D)** Enteroids isolated from WT or AMPKα1/2^−/−^mice were treated or not (“Un”) with the indicated pharmacologic agonists of AMPK (1 mM Met, metformin; 100 μM A7, A769662) for 4 h before lysis. Equal aliquots of whole cell lysates were analyzed for activation of AMPK and the SPS-pathway by immunoblotting. **(E)** Bar graphs display the change in trans-epithelial electrical resistance (TEER) across EDMs prepared from WT and AMPKα1/2^−/−^mice. Findings were analyzed by one-way ANOVA followed by multiple comparison test. Error bars represent mean ± SEM, n = 3; **P < 0.01. **(B, F)** Murine EDMs derived from WT or AMPKα 1/2^−/−^ mouse colons were prepared as described in (B), fixed, and analyzed for the integrity of the epithelial tight junctions and the SPS-pathway using anti-occludin and anti-pS245GIV, respectively, and imaged by confocal microscopy. *Left*: Images displayed are representative of three independent experiments. Arrowheads point to “burst” pattern of occludin staining at the disrupted tricellular TJs. Scale bars = 10 μm. *Right*: RGB profile plot (right) indicate the co-localization of pS245-GIV and occludin as assessed using an ImageJ plug-ins. See also Fig S3 for the effect of AMPK agonist on EDM. **(G)** Human EDMs were either untreated or treated with A-769662 (100 μM) for 16 h; fixed; stained with anti-pS245-GIV (red; a surrogate measure of the SPS-pathway), occludin (green; a *bonafide* TJ marker), and nucleus (DAPI); and analyzed by confocal microscopy. *Left*: Images displayed are representative of three independent experiments. Scale bars = 10 μm. *Right*: RGB profile plot (right) indicated the co-localization of pS245-GIV and occludin as assessed using an ImageJ plug-ins.

**Figure S1.**
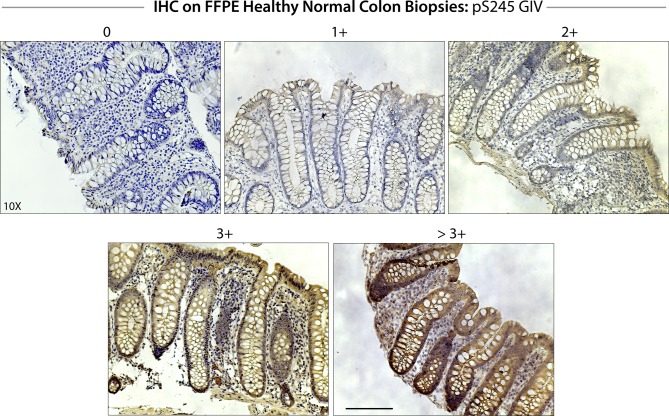
The degree of activation of the stress polarity signaling pathway in the gut lining, as determined by immunohistochemistry using anti-pS245GIV is heterogeneous among healthy patients. Scoring scheme for evaluation of the stress polarity signaling pathway in human colon biopsies. Intensity scoring for pS245-GIV staining was determined by immunohistochemistry on a scale from 0 to >3; examples of images representative of each intensity is shown. The score “0” represents the lowest degree of staining, and the score “>3+” represents the highest degree of staining.

First, we asked if the SPS-pathway is active in the epithelial lining of the human colon. To this end, we performed immunohistochemistry (IHC) using the previously validated (Aznar et al, 2016) anti-pS245-GIV antibody on formalin-fixed paraffin-embedded (FFPE) colon tissues obtained during routine colonoscopy for colon cancer screening (Fig 1A). Although all tissues were histologically confirmed as “normal,” some of them were from patients with type II diabetes on chronic therapy with metformin at doses ranging from 500 to 1,500 mg/d. We preferred the use of pS245-GIV as a “surrogate marker” of the SPS-pathway (as opposed to other global anti-phospho-AMPK antibodies) because pS245-GIV was previously determined (Aznar et al, 2016) to be the TJ-localized substrate of AMPK activity which is both necessary and sufficient for protecting TJs from stress-induced collapse. It offers a way to specifically monitor AMPK’s TJ-stabilizing effect as opposed to its general effect on cellular bioenergetics. In fact, a side-by-side comparison of the two targets showed that although pS245GIV remained associated with TJs in a sustained manner, pAMPK is activated only transiently at tricellular contact site and then shows a cytosolic staining pattern around disrupted TJs (Aznar et al, 2016). We found that although the intensity of staining varies among patients, a larger proportion of patients on metformin displayed positive staining and at a stronger intensity compared with the cohort of patients, not on metformin (Figs 1A and S1). These findings indicate that the SPS-pathway, as determined by pS245-GIV as a surrogate marker, is active in the human colon epithelium and that although its degree of activation varies considerably in the normal colon, it appears to be consistently enhanced in patients exposed to the widely prescribed AMPK-agonist metformin.

To translate the physiological implications of the observations made using polarized MDCK monolayers (Aznar et al, 2016), we used an ex vivo near-physiologic model system called the “gut-in-a-dish” (see the legend and Fig 1B). In this model, crypt-derived stem cells isolated from human or mouse colon (see the Materials and Methods section) were used to generate organoids and later differentiated into polarized enteriod-derived monolayers (EDMs). These EDMs have been validated as model systems that closely resemble the physiologic gut lining in which all cell types (enterocytes, goblet, Paneth enteroendocrine, and tuft cells) are proportionately represented (Sato et al, 2009; Miyoshi & Stappenbeck, 2013; Foulke-Abel et al, 2014; Noel et al, 2017; Yu et al, 2017). We used this “gut-in-a-dish” model to rigorously interrogate the impact of various stressors on the integrity of the gut barrier and cellular processes, using readouts illustrated in Fig 1B. Using pS245-GIV as a marker, we confirmed that the SPS-pathway is active in the EDMs, colocalized with the TJ protein, occludin, as previously shown in MDCK cells; (Aznar et al, 2016). When we treated the EDMs with metformin, the pathway was significantly enhanced—whereas immunofluorescence studies (Figs 1C-Left and S2) showed a approximately fivefold to sixfold increase in the intensity of occludin staining, immunoblots (Fig 1C-Right) showed a peak increase in pS245 GIV and pAMPK up to approximately twofold to threefold at 4 h.

**Figure S2.**
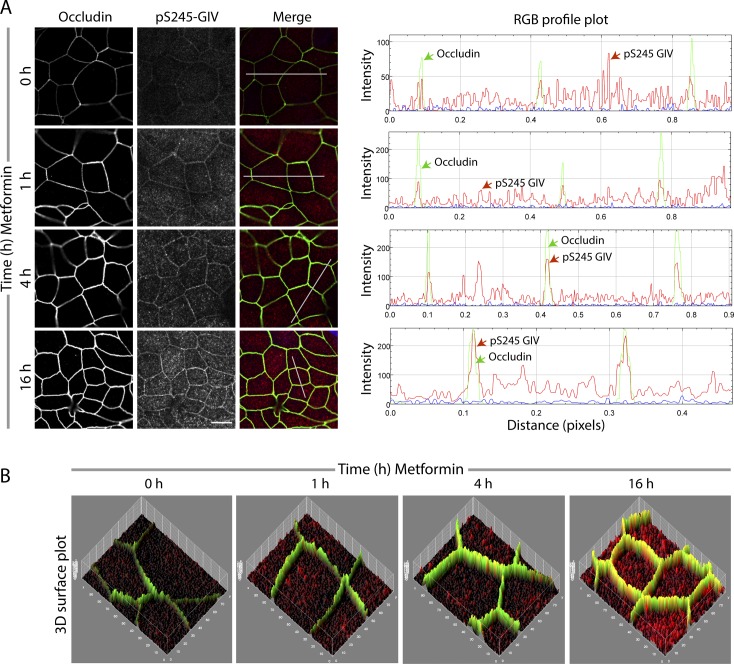
The stress polarity signaling pathway can be pharmacologically augmented with metformin in polarized murine enteroid-derived monolayers. **(A, B)** WT Murine enteroid-derived monolayers were treated with metformin (1 mM) for various time points as indicated; fixed; stained with anti-pS245-GIV (red; a surrogate measure of the stress polarity signaling-pathway), occludin (green; a bona fide tight junction marker), and nucleus (DAPI); and analyzed by confocal microscopy. **(A)** Images displayed are representative of three independent experiments (*left*). **(A, B)** Co-localization of pS245-GIV and occludin is assessed using ImageJ plug-ins: RGB profile plot (A-*right*) and 3D surface plots (B).

**Figure S3.**
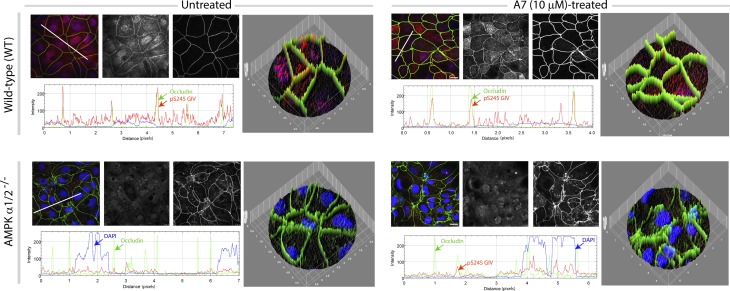
WT or AMPKá1/2^−/−^ murine enteroid-derived monolayers pretreated or not with A7 for 16 h were assessed for tight junction integrity (occludin) and stress polarity signaling pathway activation (pS245 GIV) using confocal microscopy.

Next, we asked if activation of the SPS-pathway in the gut epithelium requires the catalytic activity of AMPK. We assessed the levels of pS245-GIV in enteroids using two parallel approaches: 1) We generated enteroids from AMPKα1/2-Villin-Cre mice (generated by Benoit Viollet, Institut National de la Santé et de la Recherche Médicale (French Institute of Health and Medical Research) [INSERM]; manuscript in preparation) which lack both the α-isoforms that encode the catalytic subunits of the heterotrimeric kinase. 2) We used a well-validated direct agonist of AMPK, A-769662 (Cool et al, 2006), for which the structural basis for activation of the heterotrimer is known (Xiao et al, 2013). We found that neither metformin nor A7 could induce pS245-GIV in enteroids devoid of AMPKα1/2 (Fig 1D). Compared with EDMs from WT mice, EDMs derived from AMPKα1/2-Villin-Cre mice showed impaired barrier integrity and higher paracellular permeability, as reflected by low trans-epithelial electrical resistance (TEER; Fig 1E), absence of pS245-GIV staining at the TJs, and aberrant TJ morphology detected using occludin a bona fide TJ protein (“burst” appearance of tricellular TJs; see arrowheads, Fig 1F). Although pS245GIV was virtually absent at TJs, some nuclear speckles were observed in AMPKα1/2-Villin-Cre EDMs, the significance of which remains unknown. Furthermore, when we treated the WT or AMPKα1/2-Villin-Cre EDMs with A-769662, the SPS-pathway was enhanced in the WT EDMs, as determined by increased intensities of both occludin and pS245GIV, but the pathway could not be rescued in the AMPKα1/2-Villin-Cre EDMs (Fig S3). The AMPK agonists also activated the SPS-pathway in human EDMs (Fig 1G). Together, these results using a combination of AMPK-depleted EDMs and highly specific AMPK agonist demonstrate that the SPS-pathway, as determined by the abundance of pS245-GIV in the gut epithelium, requires the metabolic kinase AMPK, and its absence compromises the integrity of the epithelial barrier.

### The SPS-pathway protects the gut barrier against diverse stressors such as microbes and microbial products

**Figure 2.**
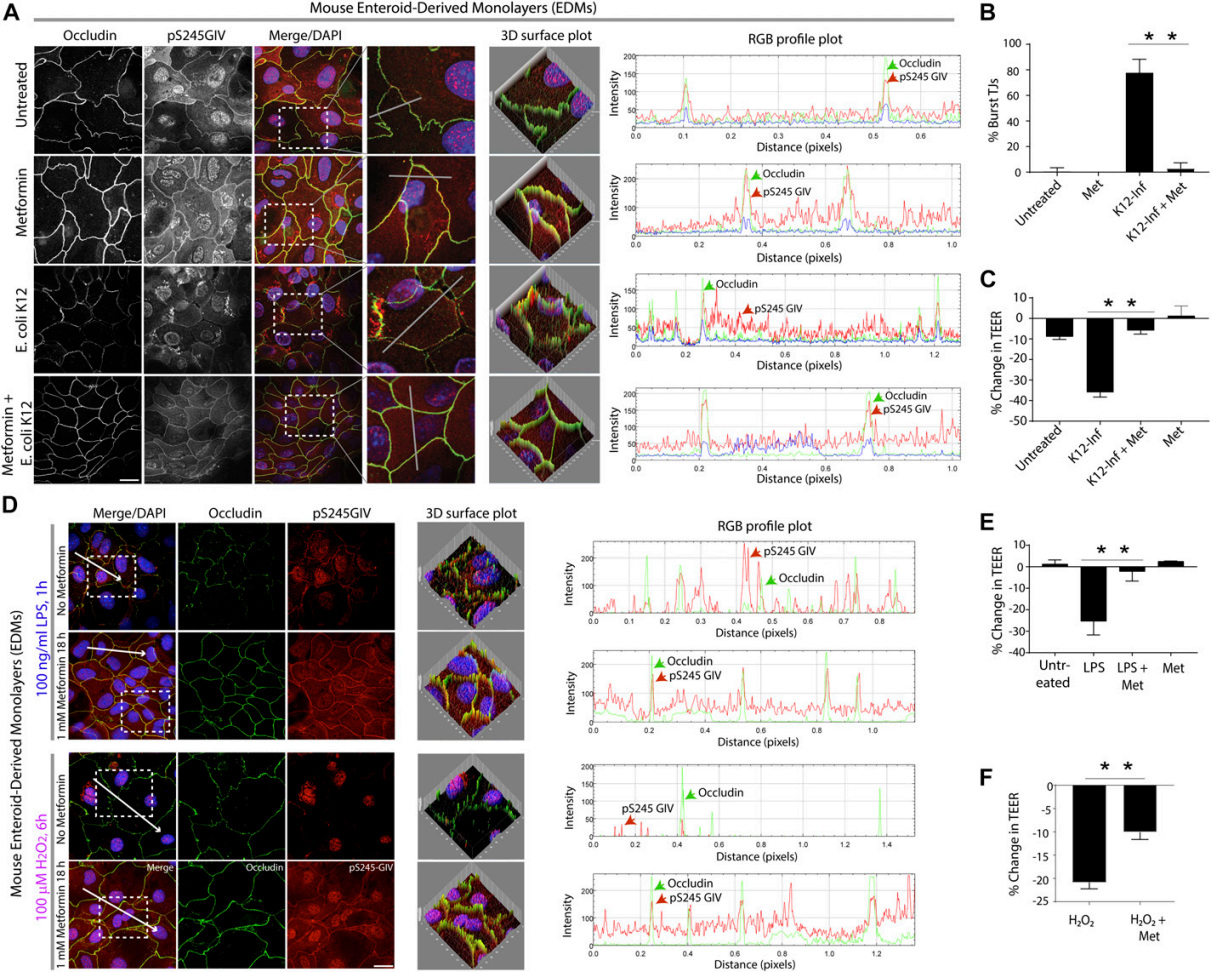
Pharmacologic augmentation of the stress polarity signaling (SPS) pathway protects the gut barrier against diverse stressors such as microbes and microbial products. **(A)** Mouse enteroid-derived monolayers (EDMs) were infected with *E. coli* K12 with or without prior exposure to metformin (1 mM for 16 h); fixed; stained with anti-pS245-GIV (red; a surrogate measure of the SPS-pathway), occludin (green; a bona fide tight junction [TJ] marker), and nucleus (DAPI); and analyzed by confocal microscopy. *Left*: Images displayed are representative of three independent experiments. **(A)** Co-localization of pS245-GIV and occludin was assessed using ImageJ plug-ins: RGB profile plot (A-*middle*) and 3D surface plots (A-*right*). Scale bars = 10 μm. **(A, B)** Bar graphs display the % of TJs that appeared broken and/or splitting (“burst”-appearing) on the y-axis encountered in 8–10 randomly chosen fields from three independent experiments in (A). Findings were analyzed by one-way ANOVA followed by multiple comparisons test. Error bars represent mean ± SEM, n = 3; **P < 0.01. **(A, C)** Bar graphs display the change in TEER across the EDMs in (A). Findings were analyzed by one-way ANOVA followed by multiple comparison test. Error bars represent mean ± SEM; n = 3; **P < 0.01. **(D)** Mouse EDMs pretreated (or not) with metformin (1 mM for 16 h) were exposed to LPS or H_2_O_2_, as indicated; fixed; stained with anti-pS245-GIV (red; a surrogate measure of the SPS-pathway), occludin (green; a bonafide TJ marker), and nucleus (DAPI); and analyzed by confocal microscopy. *Left*: Images displayed are representative of three independent experiments. Scale bars = 10 μm. *Right*: Co-localization of pS245-GIV and occludin was assessed using ImageJ plug-ins, RGB profile plot. **(D, E, F)** Bar graphs display the change in TEER of the EDMs after LPS (E) or H_2_O_2_ (F) treatment shown in (D). Findings were analyzed by one-way ANOVA followed by multiple comparisons test. Error bars represent mean ± SEM; n = 3; **P < 0.01.

Next, we used the “gut-in-a-dish” model to study the role of the SPS-pathway in EDMs under stress. Our choice of stressors included those that are physiologically encountered within the gut lumen, for example, a) live commensal microbes (*Escherichia coli*; Fig 2A–C), b) microbial outer membrane components (LPS; Fig 2D and E) and H_2_O_2_ (Fig 2D and F); the latter induces reactive oxygen species in ways that mimic the host response to dysbiosis or in response to injury or inflammation. EDMs were either treated or not with metformin followed by exposure to each stressor. EDMs were assessed for barrier integrity by analyzing the same sample by two assays: a) periodic measurements of TEER and b) immunofluorescence with pS245-GIV and occludin to assess the SPS-pathway and TJ morphology, respectively. We continued to monitor occludin as a readout of TJ morphology because this integral membrane protein allows us to not just visualize but also quantify the degree of TJ disruption (“burst” tricellular TJs where three or more cells come in contact [Furuse et al, 2014]); the tricellular TJs appear to be the regions where earliest evidence of disruption can be visualized/assessed first. Also, prior work (Aznar et al, 2016) showed that under stress, active AMPK was detected transiently at the TJs of tricellular contact, as determined by colocalization of phospho-AMPK with occludin. We found that all stressors induced barrier disruption in untreated EDMs, as determined by occludin staining (Fig 2A, B, and D) and by the observed drops in TEER (Fig 2B, C, E, and F). Pretreatment of EDMs with metformin showed SPS-pathway activation (pS245), maintenance of TJ architecture (occludin), and preservation of barrier integrity (TEER). We conclude that treatment with metformin resists barrier collapse in all stress-inducing conditions tested, implicating the barrier-protective role of the SPS-pathway in the gut epithelium.

### The SPS-pathway is suppressed in the aged gut; its loss triggers inflammation

**Figure 3.**
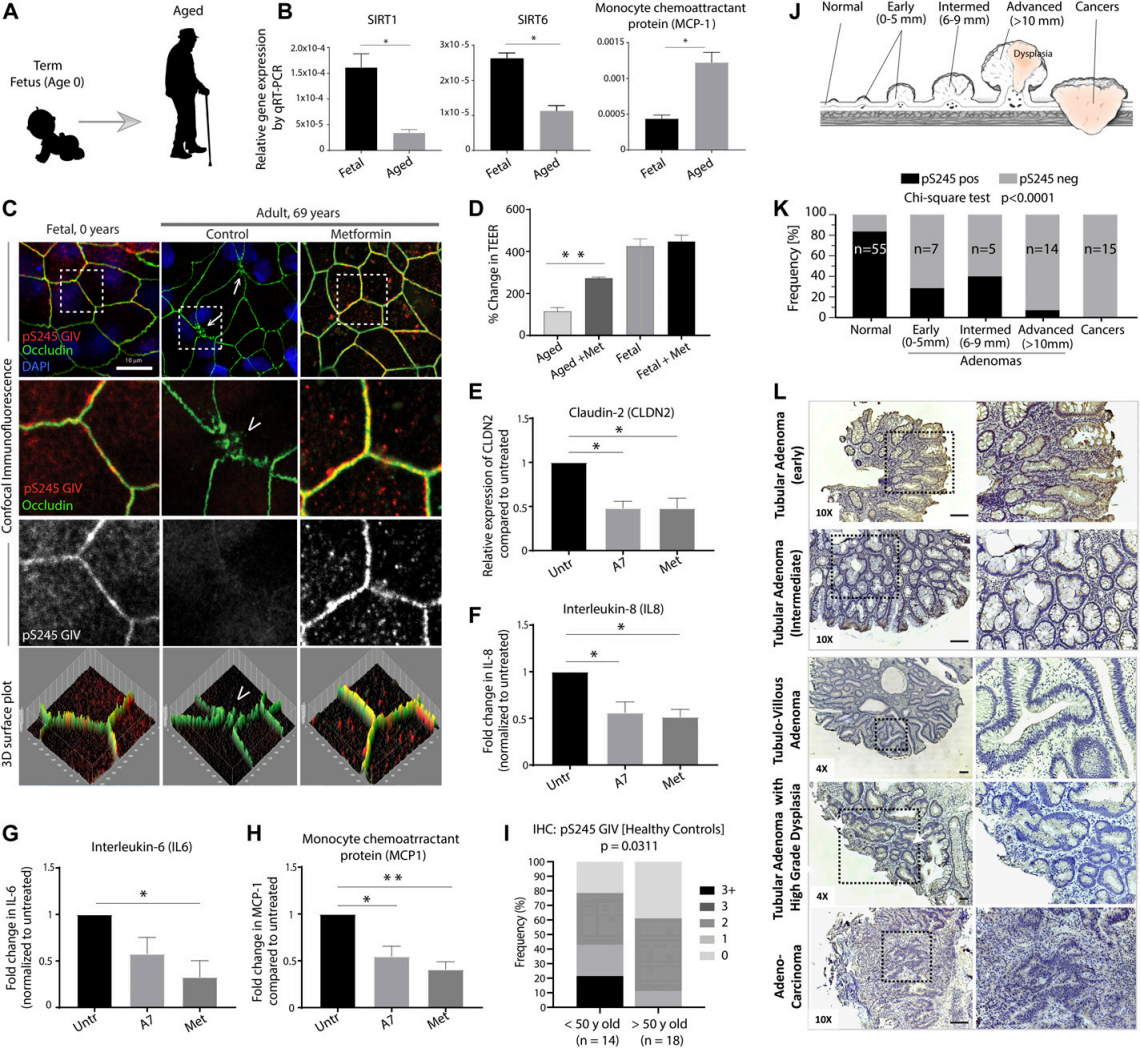
Stress polarity signaling pathway is impaired in the aging gut and during the initiation and progression of colorectal cancers. **(A, B, C, D, E, F, G, H)** Schematic showing the profile of human enteroid-derived monolayers (EDMs) used in panels (B, C, D, E, F, G, H). Term fetus is indicated as age 0 yr. **(B)** Fetal (0 yr; n = 3 subjects) and aged (∼50–70-yr old; n = 3 subjects) human EDMs were analyzed for aging molecules, Sirtuins (SIRT)1 and 6, and MCP-1 by qRT-PCR. Data displayed as mean ± SEM from three independent experiments. Findings were analyzed by one-way ANOVA followed by multiple comparisons test. * = *P* < 0.05. **(B, C)** EDMs in (B) were pretreated or not with metformin (1 mM for 16 h); fixed; stained with anti-pS245-GIV (red; a surrogate measure of the stress polarity signaling-pathway), occludin (green; a *bonafide* tight junction marker), and nucleus (DAPI); and analyzed by confocal microscopy. *Above*: Images displayed are representative of three independent experiments. Scale bars = 10 μm. Co-localization of pS245-GIV and occludin was assessed using ImageJ plug-ins and below, 3D surface plots. **(C, D)** Bar graphs display the change in TEER of the EDMs in (C). Findings were analyzed by two-way ANOVA followed by multiple comparisons test. Error bars represent mean ± SEM; n = 3; **P < 0.01. **(E, F, G, H)** The aged (69-yr-old) human EDMs were treated or not (“Untr”) with metformin (1 mM) or A7 (A769662; 100 μM) for 16 h before analyzing them for the levels of expression of CLDN2 (claudin-2), IL-8, IL-6, and MCP-1 by qRT-PCR. Data displayed as mean ± SEM from three independent experiments normalized to untreated control. Findings were analyzed by two-way ANOVA followed by multiple comparisons test. * = *P* < 0.05; ** = *P* < 0.01. **(I)** FFPE human colon tissues representing young (<50 yr; n = 14) or aged (>50 yr; n = 18) were analyzed for pS245-GIV by immunohistochemistry, and the staining intensity in the epithelium was scored (see Fig 1 Supplement 2). Bar graph displays the proportion of patients in each group with varying intensities of staining. Two-sided Fisher’s exact test was used to calculate significance. **(J, K, L)** FFPE human colon tissues representing normal, adenomas, and carcinomas (schematic, J) were analyzed for pS245-GIV by immunohistochemistry. Bar graph (K) displays the proportion of samples in each category that stained positive. Two-sided Fisher’s exact test was used to calculate significance. **(L)** Images representative of each category are displayed (L). Panels on the right are magnified boxed areas of the images on the left. Scale bar = 100 μm.

Among the various organ systems that decline during aging, dysfunction of the intestinal barrier has been correlated with increasing age in a variety of species. For example, dysfunction of the intestinal barrier predicts impending death in individual flies regardless of their chronological age (Rera et al, 2012). Much like humans, dysregulation of barrier in these flies shows an age-related increase in immunity-related gene expression (e.g., IL-6) (Rera et al, 2012). Evidence also shows that intestinal barrier dysfunction during aging is conserved in worms (*Caenorhabditis elegans*), fish (*Danio rerio*) (Tricoire & Rera, 2015; Dambroise et al, 2016), and mammals (rats [Katz et al, 1987] and baboons [Tran and Greenwood-Van Meerveld, 2013]), thus raising the possibility that it may also be the case in humans. To investigate this, we first characterized human enteroids derived from term male fetuses and “normal” colon biopsies from elderly male subjects (Fig 3A) for transcript expression levels of SIRTs 1 and 6, two key aging/longevity related families of proteins with either mono-ADP-ribosyltransferase or deacylase activity, that is, sirtuins because previous lifespan studies carried out using yeast, worms, and flies as model organisms have demonstrated that sirtuins are evolutionarily conserved mediators of longevity (Kaeberlein et al, 1999; Tissenbaum & Guarente, 2001; Rogina & Helfand, 2004). Both SIRTs have been shown to regulate inflammation in the gut (Akimova et al, 2014; Lo Sasso et al, 2014; Wellman et al, 2017; Zhang et al, 2018). We found that compared with fetal EDMs, aged EDMs expressed less SIRT1 and 6 (Fig 3B-Left, Middle), consistent with the previous observations of their decline in the aged gut (Liu et al, 2017a; Igarashi et al, 2019). Because age-associated changes in gut microbiome composition is correlated with increases in the pro-inflammatory marker serum monocyte chemoattractant protein (MCP-1) (Conley et al, 2016), which in turn has been implicated in aging-related macrophage dysfunction (Thevaranjan et al, 2017), we analyzed this cytokine in the aged EDMs. We found that MCP-1 transcript levels were increased in aged EDMs relative to fetal EMDs (Fig 3B-Right).

Increased intestinal leakiness in the aged gut has been documented (Valentini et al, 2014). Therefore, next, we analyzed the barrier integrity of and the status of the SPS-pathway in the aged EDM. We found by confocal microscopy that TJs were more frequently disrupted in the aged compared with fetal EDMs (Fig 3C; occludin signal) and that the SPS-pathway is suppressed (Fig 3C; pS245-GIV signal was virtually undetectable). Treatment of the aged EDMs with metformin restored the SPS-pathway, repaired the “burst” TJs (visualized by occludin staining) (Fig 3C), and increased TEER (Fig 3D). Pharmacologic activation of the SPS-pathway either with metformin or the AMPK-specific activator, A-769662 (Fig 3C–H), reduced the leaky TJ-protein, claudin-2 (CLDN2) (Fig 3E), and suppressed proinflammatory cytokines (IL8, IL6, and MCP1) (Fig 3F–H). These findings indicate that pharmacologic activation of AMPK and restoration of the SPS-pathway is sufficient to reduce pro-inflammatory cytokines that are seen in the aged EDMs. Finally, we confirmed that the SPS-pathway is indeed suppressed in the aged gut lining, as determined by IHC on FFPE colon tissues obtained during routine colonoscopy from patients representative of various age groups (Fig 3I). Taken together, we conclude that the SPS-pathway is suppressed in the aged gut epithelium, and such suppression is permissive to a pro-inflammatory program.

### The SPS-pathway is suppressed during colorectal cancer (CRC) initiation and progression

Previously, Aznar et al (2016) indicated the tumor-suppressive property of the SPS-pathway. Here, we investigated the activation status of the tumor-suppressive SPS-pathway during CRC initiation and progression. Because TJ-localized pS245-GIV serves as a functional molecular marker that reflects the activation state of the SPS-pathway, we carried out IHC with anti-pS245-GIV on FFPE colon tissues from various stages of normal-to-cancer progression in the colon (Fig 3J). Phospho-S245-GIV was undetectable in almost all advanced adenomas and all CRCs analyzed (Fig 3K and L), indicating that the SPS-pathway is lost or silenced during CRC progression. This finding is consistent with our previous findings (Aznar et al, 2016) demonstrating the tumor-suppressive property of the SPS-pathway in 3D-cultured DLD1 CRC cell lines. It is important to note that the pattern of pS245GIV staining is strikingly different from the levels of total GIV during CRC initiation and progression; we have previously shown that GIV mRNA and protein generally decreases first during polyp formation and progression and subsequently increases during CRC progression to metastasis (Ghosh et al, 2010). We and others have shown that increased levels of GIV in established CRCs correlate with aggressive features (Garcia-Marcos et al, 2011b; Jun et al, 2013; Aznar et al, 2016; Barbazan et al, 2016; Ghosh et al, 2016; Lu et al, 2018), for example, shorter metastasis-free survival, chemoresistance, and stemness, largely attributed to its ability to scaffold multi-receptor signaling cascades and enhance them via G protein intermediates to trigger epithelial mesenchymal transition (reviewed in Ghosh (2015)). Taken together, the loss of pS245 GIV early during polyp to CRC progression underscores how GIV may serve as a tumor suppressor when localized to TJs in the polarized normal epithelium before it assumes its role as an enhancer of epithelial–mesenchymal transition and stemness in established CRCs.

### Conclusion

**Figure 4.**
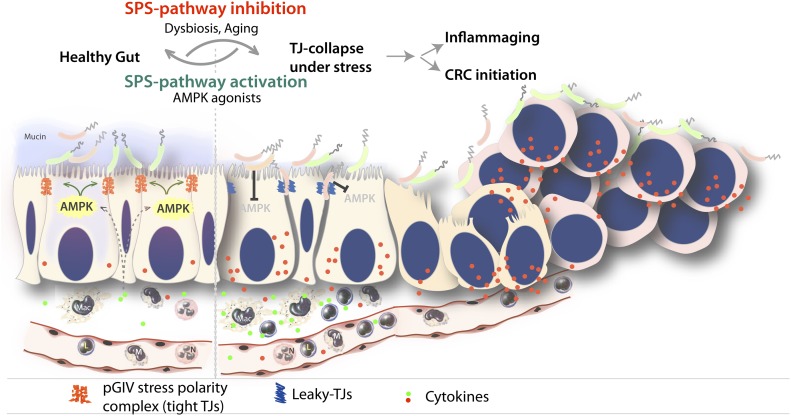
Summary of findings and working model. Summary and proposed working model for the role of the stress polarity signaling (SPS) pathway in the gut barrier. Genetic, epigenetic, or dysbiosis-induced inhibition of the SPS-pathway leading to stress-induced tight junction-collapse and loss of cell polarity. Loss of the SPS-pathway (in aging and during cancer initiation) is accompanied by collapse of epithelial tight junctions and loss of cell polarity, which is permissive to a gene expression signature that promotes leakiness of the gut barrier (high claudin-2) and inflammation (MCP1, IL8). Pharmacologic augmentation of the SPS-pathway with AMPK agonists may resist and reverse the above.

The importance of the gut barrier has gained so much traction in the past decade that it has ushered in the dawn of “barriology” (Tsukita et al, 2008). The current thinking in the field of “barriology” is that chronic systemic endotoxemia, due to a compromised gut barrier in the setting of stress, impacts multiple diseases. Microbial dysbiosis is known to trigger major cellular programs, for example, loosening of cell–cell junctions, loss of cell polarity, and chronic inflammation, all of which coordinately fuel the progression of these chronic diseases. This work reveals the physiologic importance and therapeutic potential of the SPS-pathway to resist and reset gut microbe triggered programs by fortifying epithelial TJs (see Summary and working model; Fig 4). Using the combined synergy of patient-derived tissues and mouse and human organoid-based models, we showed that the SPS-pathway serves as a protective host response that is compromised in the aged gut and early during the initiation of colon cancers.

This work highlights the SPS-pathway as an “actionable pathway” in the gut barrier. The abundance of TJ-localized pS245-GIV serves as a reliable marker of the SPS-pathway and a surrogate measure of the integrity of the gut barrier. These are important findings because despite decades of research showing the importance of the gut barrier in health and disease and discovery of plausible targets (Cunningham & Turner, 2012), our ability to detect and fix a leaky gut barrier is not currently a practical option in clinical practice. By demonstrating direct and specific AMPK-agonists as effective augmentors of gut barrier integrity and by validating pS245-GIV as a reliable molecular marker to track such pharmacologic augmentation, this work translates the therapeutic and diagnostic potential of the fundamental discoveries made by Aznar et al (2016).

With regard to the impact of our findings in human diseases, because chronic low-grade inflammatory status in the aging gut has led to coining of the term “*inflammaging*” (Giunta, 2006; Franceschi, 2007; Franceschi et al, 2007; Goto, 2008; Minciullo et al, 2016; Manabe, 2017), our findings suggest that activation of the SPS-pathway may serve as a strategy to combat this entity. Because the SPS-pathway was initially characterized as a tumor-suppressive pathway, and we have now shown that this pathway is silenced early during CRC initiation, these findings suggest that activation of the SPS-pathway may serve as a strategy to prevent polyp-to-cancer progression in the colon. Our findings also raise the possibility that the SPS-pathway may affect a variety of non-oncologic diseases that are associated with increased intestinal permeability (reviewed in Bischoff et al (2014)) such as chronic gastrointestinal inflammation, Alzheimer’s, Parkinson’s, multiple sclerosis, autism, chronic heart failure, and obesity and metabolic diseases. All these diseases are characterized by systemic inflammation due to chronic endotoxemia that might be triggered by the translocation of endotoxins from the gut lumen into the host circulation.

By demonstrating the therapeutic potential of metformin as an activator of the SPS-pathway, our findings revisit the mechanism of action of this first-line treatment for type II diabetes. Although metformin (Glucophage) is now the most widely prescribed type II diabetes drug in the world for its ability to reduce blood glucose by activating the LKB1-AMPK pathway (Shaw et al, 2005) and inhibiting hepatic gluconeogenesis (reviewed in Shackelford and Shaw (2009)), it is also known to exert other effects in an AMPK-dependent manner: (i) it stabilizes cell–cell junctions and protects barrier functions of both epithelial and endothelial monolayers in the setting of a variety of pathologic stressors and (ii) it suppresses the growth of a variety of tumor cells and embryonic stem cells in culture and tumor xenografts in mice (reviewed in Shackelford and Shaw (2009)). Numerous studies using the AMPK-activator, metformin, squarely implicate the AMPK-dependent stress polarity pathway as a major therapeutic target in these metabolic disorders (Everard et al, 2013; Buse et al, 2016; Zhou et al, 2016). metformin administration enhances gut barrier integrity, attenuates endotoxemia, and enhances insulin signaling in high-fat fed mice, which accounts for the beneficial effects of metformin on glucose metabolism, enhanced metabolic insulin response, and reduced oxidative stress in liver and muscle of the mice (Zhou et al, 2016). It is possible that the observed anti-ageing properties of metformin (via multiple widely pleiotropic effects reviewed in Barzilai et al (2016)), as in the case of obesity and diabetes, may begin by preserving the gut barrier function, thereby reducing age-related inflammation and metabolic derangements. If so, metformin is expected to act via the AMPK-GIV SPS-pathway to resist aging-related increase in gut permeability. Ongoing clinical trials approved by the Food and Drug Administration (such as Targeting Ageing with Metformin; TAME) are likely to provide the best opportunity to investigate these possibilities. In fact, one clinical trial already hints at that. Using a delayed release formulation of metformin (metformin DR, which is designed to target the lower bowel and limit absorption into the blood), it has been shown that metformin works largely in the colon; despite the reduced levels of absorption of metformin DR, this formulation was effective in lowering blood glucose (Buse et al, 2016). Our findings in human organoid models not only bridge the gap between murine studies and the clinical trials on diabetics but also pinpoint a mechanism via which metformin’s ability to activate AMPK may serve as a therapeutic strategy to reinforce the gut barrier.

In closing, our findings underscore the importance of the SPS-pathway in the gut epithelium for the maintenance of cell polarity and the epithelial barrier. By showing previously how the SPS-pathway serves as a tumor suppressive pathway (Aznar et al, 2016) and showing here how the SPS-pathway suppresses proinflammatory cytokines in the aging gut (“inflammaging”), we have linked this specialized polarity pathway polarity to epithelium-driven pathophysiologic processes. By demonstrating here that the SPS-pathway is suppressed during aging and during CRC initiation, we implicate a loss of this pathway as a bona fide pathophysiologic component of aging and an early event during oncogenesis. Insights gained also reveal the diagnostic and therapeutic potential of the SPS-pathway—its pharmacologic augmentation could be exploited as a strategy for resisting aging, CRC chemoprevention, and presumably for combating a myriad of other diseases that are characterized by chronic inflammation and endotoxemia arising from a leaky gut barrier.

## Materials and Methods

### Reagents and antibodies

Unless otherwise indicated, all reagents were of analytical grade and obtained from Sigma-Aldrich. Custom-designed oligos were obtained from Valuegene. Antibodies against GIV that were used in this work include rabbit serum anti-GIV coiled-coil immunoglobulin G (GIV-ccAb for immunoblotting only) (Le-Niculescu et al, 2005) and affinity-purified GIV ccAb (Cat. no. ABT80; from EMD Millipore for immunoblotting). Mouse mAbs against anti-phospho-(p; Cell Signaling Technology) and total (t; Abcam) AMPK, and tubulin (Sigma-Aldrich) were purchased from commercial sources. Rabbit polyclonal antibodies against phospho-S245 GIV were generated commercially by 21st Century Biochemicals and extensively validated previously (Aznar et al, 2016). DAPI and antimouse Alexa Fluor 594–coupled goat secondary antibody for immunofluorescence were purchased from Invitrogen. Goat antirabbit and goat antimouse Alexa Fluor 680 or IRDye 800 F(ab′)_2_ for immunoblotting were from LI-COR Biosciences.

### Animal studies

C57BL/6 mice of 6–8 wk age from both genders were used to isolate intestinal crypts. Animals were bred, housed, used for all the experiments, and euthanized according to the University of California San Diego Institutional Animal Care and Use Committee policies under the animal protocol number S18086. All methods were carried out in accordance with relevant guidelines and regulations and the experimental protocols were approved by institutional policies and reviewed by the licensing committee.

### Human subjects

For immunohistochemical analysis of human tissue specimens, archived FFPE human colonic biopsies from healthy controls or patients with adenomas and/or carcinomas were obtained from the Gastroenterology Division, VA San Diego Healthcare System, following the protocol approved by the Human Research Protection Program Institutional Review Board (Project ID# 1132632).

For the purpose of generating adult healthy enteroids, a fresh biopsy was prospectively collected using small forceps from healthy subjects undergoing routine colonoscopy for colon cancer screening. For all the deidentified human subjects, information including age, ethnicity, gender, previous history of disease, and medication was collected from the chart following the rules of HIPAA. Each human participant was recruited to the study following an approved human research protocol and signed a consent form approved by the Human Research Protection Program at the University of California, San Diego, to agree that the colonic specimens from their colonoscopy will be used to generate an enteroid line for functional studies.

### Isolation, expansion, and culture of organoids from mouse and human colons

Intestinal crypts were isolated from the colonic tissue specimen by digesting with collagenase type I (2 mg/ml; Life Technologies Corporation) and cultured in stem cell–enriched conditioned media (CM) with WNT 3a, R-spondin, and noggin (Sato et al, 2009; Miyoshi & Stappenbeck, 2013; Mahe et al, 2015). Briefly, the crypts after digestion with collagenase were filtered with a cell strainer and washed with the medium (DMEM/F12 with Hepes, 10% FBS). After adding collagenase I solution containing gentamicin (50 μg/ml; Life Technologies Corporation) and mixing thoroughly, the plate was incubated at 37°C inside a CO_2_ incubator for 10 min, with vigorous pipetting between incubations and monitoring constantly by light microscopy to confirm by direct observation the dislodgement of the intestinal crypts from the tissues. The collagenase was inactivated with media and filtered using a 70-μm cell strainer over a 50-ml centrifuge tube. Filtered tissue was spun down at 200*g* for 5 min, and the media was aspirated. The epithelial units were suspended in Matrigel (basement membrane matrix; Cat. no. 356235; Corning Inc.). Cell-Matrigel suspension (15 μl) was placed at the center of the 24-well plate on ice and placed on the incubator upside-down for polymerization. After 10 min, 500 μl of 50% CM was added. CM was prepared from L-WRN cells (CRL-3276; ATCC, from the laboratory of Thaddeus S. Stappenbeck [Miyoshi & Stappenbeck, 2013]) with Wnt3a, R-spondin, and noggin. Y27632 (ROCK inhibitor, 10 μM) and SB431542 (an inhibitor for TGF-β type I receptor, 10 μM) were added to the medium. For the human enteroids, media and supplements were obtained commercially (Cell Applications Inc.), and a proprietary cocktail was added to the above medium. The medium was changed every 2–3 d and the enteroids were expanded and frozen in liquid nitrogen.

AMPK KO enteroids were isolated from KO AMPK mice specifically deleted in the intestinal epithelial cells; these mice were generated at INSERM by crossing Villin-Cre mice with AMPKα1^f1/fl^/α2^fl/fl^ mice (manuscript in preparation). As for human studies, ∼4–6 healthy human individuals were used across the experiments testing their junctional integrity and response to AMPK agonists.

### Preparation of EDMs

EDMs were prepared from colon spheroids as previously described (den Hartog et al, 2016; Suarez et al, 2018 *Preprint*). Briefly, the spheroids were trypsinized and the isolated cells were filtered, counted, resuspended in 5% CM, and plated with Matrigel (1:40 dilution) in the apical part of 0.4-μm polyester membrane transwell (Cat. no. 3470; Corning) at the density of 2 × 10^5^ cell/well. In some cases, the EDMs were also differentiated for 2 d in advanced DMEM/F12 media without Wnt3a but with R-spondin, noggin, B27 and N2 supplements, and 10 μM ROCK inhibitor (Sato et al, 2009). As expected, this results in a marked reduction in the expression of the stemness marker Lgr5 in EDMs (Sato et al, 2009).

### Bacterial culture

*E. coli* K12 strain DH10B, pTransSacB (PTA5105), was obtained from ATCC; the bacteria was cultured on LB agar and LB broth and used to infect EDM at moi 100.

### Infection of EDMs with live microbes

Mouse and human EDMs were prepared as described in the previous section. EDMs were differentiated for 2 d before treatment with various chemical activators of AMPK, that is, metformin (1 mM) and, A-769662 (100 μM) for 16 h. Cultures were then challenged with either microbial products, that is, LPS (500 ng/ml) and H_2_O_2_ (100 μM) or live microbes (*E. coli* K12). On the day of infection, the medium in the basolateral part was also replaced with fresh 5% medium. Trans-epithelial electrical resistance (TEER) was measured using an epithelial voltohmmeter Millicell-ERS resistance meter (Millipore) before and at specific time points after the treatment (0, 4, 8, and 24 h). The supernatant was collected from the basolateral and apical part of the transwell for cytokine analysis, and the cells were collected for RNA extraction followed by expression of target genes by qRT-PCR.

### Immunofluorescence

Mouse and human EDM were fixed with cold methanol at −20°C for 20 min, washed once with phosphate-buffered saline and blocked with IF buffer (0.1% Triton TX-100 and 2 mg/ml BSA, in PBS) for 1 h. The samples were then incubated with primary and then secondary antibodies as described previously (Ghosh et al, 2008). Dilutions of antibodies and reagents were as follows: anti-phospho-Ser245-GIV (pS245-GIV; 1/250), anti-occludin (1/250), anti-claudin-2-1 (1/250), DAPI (1:1,000), and goat antimouse Alexa Flour (488 and 594)–conjugated antibodies (1:500). Images were acquired using a Leica CTR4000 Confocal Microscope with a 63× objective. Z-stack images were obtained by imaging ∼4-μm-thick sections of cells in all channels. Cross-section images were obtained by automatic layering of individual slices from each Z-stack. Red-green-blue (RGB) graphic profiles were created by analyzing the distribution and intensity of pixels of these colors along a chosen line using Image J software. All individual images were processed using Image J software and assembled for presentation using Photoshop and Illustrator software (Adobe). Quantification of burst tight junctions was performed by manually counting the number of total and burst tri-cellular junctions in 8–10 randomly chosen fields in each of three independent experiments. Results are expressed as the frequency of burst TJs, and a one-way ANOVA analysis was used to determine significance.

### Immunoblotting

For immunoblotting, protein samples were separated by SDS–PAGE and transferred to Polyvinylidene fluoride (PVDF) membranes (Millipore). The membranes were blocked with PBS supplemented with 5% nonfat milk (or with 5% BSA when probing for phosphorylated proteins) before incubation with primary antibodies. Infrared imaging with two-color detection and band densitometry quantifications were performed using a Li-Cor Odyssey imaging system exactly as performed previously (Garcia-Marcos et al, 2010, 2011a, 2011c, 2012; Ghosh et al, 2010). All Odyssey images were processed using ImageJ software (NIH) and assembled into figure panels using Photoshop and Illustrator software (Adobe).

### Immunohistochemistry (IHC)

FFPE tissue sections of 4 μm thickness were cut and placed on glass slides coated with poly-L-lysine, followed by deparaffinization and hydration. Heat-induced epitope retrieval was performed using citrate buffer (pH 6) in a pressure cooker. Tissue sections were incubated with 0.3% hydrogen peroxidase for 15 min to block endogenous peroxidase activity, followed by incubation with primary antibodies for 30 min in a humidified chamber at room temperature. Antibodies used for immunostaining were anti-pS245 GIV (1:50, antirabbit antibody) and anti-AMPKα2 (1:50, antirabbit; Abcam). Immunostaining was visualized with a labeled streptavidin–biotin using 3,3′-diaminobenzidine as a chromogen and counterstained with hematoxylin. The samples were quantitatively analyzed and scored based on the intensity of staining using the following scale; 0–3, where 0 = no staining, 1 = light brown, 2 = brown, and 3 = dark brown. Data are expressed as frequency of staining score, and a chi-squared test was used to determine significance.

### Data reproducibility, rigor

Each finding showcased here represents at least 3–5 independent repeats of experiments conducted on separate days. For ELISA and qPCR, results represent 2–3 technical repeats on each experiment, for a total of n = ∼3–5 independent experiments. Where immunofluorescence images are shown, representative images from randomly chosen fields are presented.

### Statistical analyses

Data are expressed as the Mean ± SEM. Statistical significance was assessed with the *t* test. Statistical significance between datasets with three or more experimental groups was determined using one-way ANOVA including a Tukey’s test for multiple comparisons. For all tests, a *P*-value of 0.05 was used as the cutoff to determine significance (*P < 0.05, **P < 0.01, ***P < 0.001, and ****P < 0.0001). All experiments were repeated a least three times, and *P*-values are indicated in each figure. All statistical analyses were performed using GraphPad prism 6.1.

## Supplementary Material

Reviewer comments
